# Effects of a soybean milk product on feto-neonatal development in rats

**DOI:** 10.7555/JBR.31.20170067

**Published:** 2018-01-26

**Authors:** Eun Suk An, Dong-Sun Park, Young-Hwan Ban, Jieun Choi, Da Woom Seo, Yoon Bok Lee, Mi Yae Shon, Ehn-Kyoung Choi, Yun-Bae Kim

**Affiliations:** 1. College of Veterinary Medicine and Veterinary Medical Center, Chungbuk National University, Cheongju 28644, Republic of Korea; 2. Central Research Institute, Dr. Chung's Food Co. Ltd., Cheongju 28446, Republic of Korea; 3. International Ginseng and Herb Research Institute, Geumsan 312804, Republic of Korea.

**Keywords:** soybean milk, feto-neonatal development, reproductive function, sperm quality

## Abstract

Since estrogenic pollutants and phytoestrogens can cause the disorder of the reproductive system, the effects of a soybean milk product (Vegemil^®^ containing 162 ppm isoflavones) on the feto-neonatal development, including male reproductive function, were investigated. Pregnant rats were fed the soybean milk (5% or 100% in drinking water) from gestational day (GD) 6 to parturition or to post-natal day (PND) 56. Specifically, the rats were divided into 4 groups: the control group (drinking water), the GD5% group (5% soybean milk during only the GD period), the GD-PND5% group (5% soybean milk during the GD and PND periods), and the GD-PND100% group (100% soybean milk instead of water during the GD and PND periods). During the gestational, lactational, and developmental periods, the reproductive and developmental parameters of dams and offspring were observed. Feeding soybean milk did not affect the birth and physical development of both male and female offspring. At PND57, the weights of the testes and epididymides of F1 males significantly increased by feeding a high concentration of the soybean milk (GD-PND100%). In addition, feeding of the soybean milk during both the GD and PND periods (GD-PND5% and GD-PND100%) enhanced the sperm counts and motility. The results indicate that soybean milk is safe for embryos, fetuses, and offspring, and improves the post-generational development of male reproductive function.

## Introduction

Since phytoestrogens were found to exert beneficial effects on the improvement of menopausal symptoms and reduction of cancer incidence^[1– 3]^, studies on flavonoids rich in beans have been carried out for a long time. The estrogenic isoflavones including genistein, daidzein, and glycitein display their effects through binding to estrogen receptors (ER)^[4]^.

By contrast, estrogenic chemicals as endocrine disruptors are well known to cause regression and dysfunction of reproductive organs, growth failure, cancers, and so on, in ecosystem and human bodies^[5– 6]^. Although phytoestrogens are believed to be relatively safe as compared to long-lasting estrogenic chemicals and innate 17β-estradiol^[7– 8]^, over-dosage and long-term use of isoflavones can also influence the endocrine system^[9– 10]^. Thus, the adverse effects of isoflavones remain controversial^[11]^.

Indeed, it was demonstrated that isoflavones adversely affect the reproductive organ development and cause bleeding in male animals^[12– 13]^. Chavarro *et al.* explained that ingestion of soybean foods renders the isoflavones to interrupt the activity of body hormones stimulating spermatogenesis, leading to a decrease in sperm number^[14]^. It was also suggested that abnormal reproductive organs could be developed in male offspring of dam taking foods containing genistein during the gestational and lactational periods and that disorders could be discovered as the male pups grow^[15]^. In another study, decreased testis weights and delayed pubertal spermatogenesis in male rats were observed by exposing to genistein during the neonatal period^[16]^. In addition, repeated treatment of genistein to male mice during the juvenile period caused Leydig cell hyperplasia and decreased sperm counts, although the motility of sperms increased^[7]^.

However, the effects of soybean ingestion on the male reproductive system and development of pubs remain unclear in many aspects, including the concentration of isoflavones in diet, exposure route (ingestion *via* meals or direct injection), adaptation of embryos, fetuses, and offsprings, exposure time and duration (during the gestational, lactational or juvenile periods), and so on^[7– 8]^. For instance, soybean milks are routinely prescribed for pre-term infants in pediatric clinics, as well as for babies as infant formula. Since soybean milks contain estrogenic ingredients (phytoestrogens) higher than mother's and cow's milks, it is necessary to check their safety for post-generational development. In the present study, we investigated the physical and sexual developments of offspring following feeding of Vegemil^®^, a soybean milk, from the embryonic to developmental stages.

## Materials and methods

### Animals

Twelve-week-old male and female Sprague-Dawley rats were purchased from Orient-Bio (Seongnam, Korea) and maintained at the constant temperature (22±1°C), relative humidity (55±10%), and photoperiod (12-hours light/dark cycle). The animals were fed standard rodent chow and purified water *ad libitum*. After the male and female rats were mated, the day when a vaginal plug was observed from females was defined as gestational day (GD) 0. All experimental procedures were approved and carried out in accordance with the Institutional Animal Care and Use Committee of the Laboratory Animal Research Center at Chungbuk National University, Korea.

### Soybean milk and treatment

Vegemil^®^, a soybean milk, was obtained from Dr. Chung's Food Co., Ltd. (Cheongju, Korea). The soybean milk (/g) contained 30 mg proteins, 35 mg fats, 40 mg carbohydrates (including 2.7 µg soybean oligosaccharides and 7 mg dietary fibers), 86.20 µg vitamins, 8.25 µg niacin, and 13.55 µg ions and electrolytes. The contents of isoflavones and their derivatives amounted to 162 ppm, in which glycoside form of isoflavones was predominant: i.e., the ratio of aglycone (such as genistein, daidzein, and glycitein) to glycoside (including genistin, daidzin, glycitin, etc.) isoflavones was 1 to 87. More specifically, the ratio of total isoflavones in Vegemil^®^ was 1 : 84 : 0.3 : 3 for aglycones : glycosides : acetylglucosides : malonylglucosides.

The pregnant female animals were randomly divided into four groups (*n* = 8/group). Control group animals (Group 1) were given normal purified water. In Group 2 (GD5%), the rats were fed water containing 5% soybean milk (Vegemil^®^) from GD6 (implantation date) to parturition date. Group 3 (GD-PND5%) and Group 4 (GD-PND100%) were given 5% and 100% of soybean milk from GD6 to PND56, respectively. The waters containing soybean milk were freshly prepared every day.

### Developmental parameters

During the entire experimental period, bodyweights of dams and offspring were recorded. Duration of pregnancy, as well as stillbirth and survival rates of offspring, was investigated. In addition, physical and sexual development parameters, including the dates of abdominal hair growth, incisor teeth eruption, eyelid opening, testicular descent, and vaginal opening, were examined. At PND57, the offspring were sacrificed and the weights of reproductive organs (testes, epididymides, prostates, seminal vesicles, uterus, and ovaries) were weighed.

### Microscopic examination

At PND57, the animals were sacrificed under deep anesthesia with diethyl ether, the male reproductive organs, including the testes, epididymides, prostates, and seminal vesicles, were removed. After a formal tissue processing, paraffin-embedded sections were stained with hematoxylin-eosin and examined under a light microscope.

### Sperm counts and motility

The right testis and epididymis of each rat were homogenized at 8,000 rpm for 2 minutes and sonicated at 4°C for 3 minutes to obtain homogenization-resistant sperm heads. The number of sperm heads was counted with a hemacytometer^[17– 18]^.

The left epididymis was dissected into a Petri dish containing M-199 media and incubated at 37°C for 10 minutes. After removing tissue debris, an aliquot of sperm was loaded on a slide glass and sperm motility was examined under a light microscope^[17– 18]^. Separately, an aliquot of sperm was stained with the same volume of 1% eosin, smeared on a slide glass, and examined for deformity of sperm.

### Statistical analysis

The results are expressed as means±SD. Tests of significance were performed using Duncan's multiple-range test after one-way analysis of variance (ANOVA), with *P*<0.05 as the criterion of significance.

## Results

**Fig.1:**
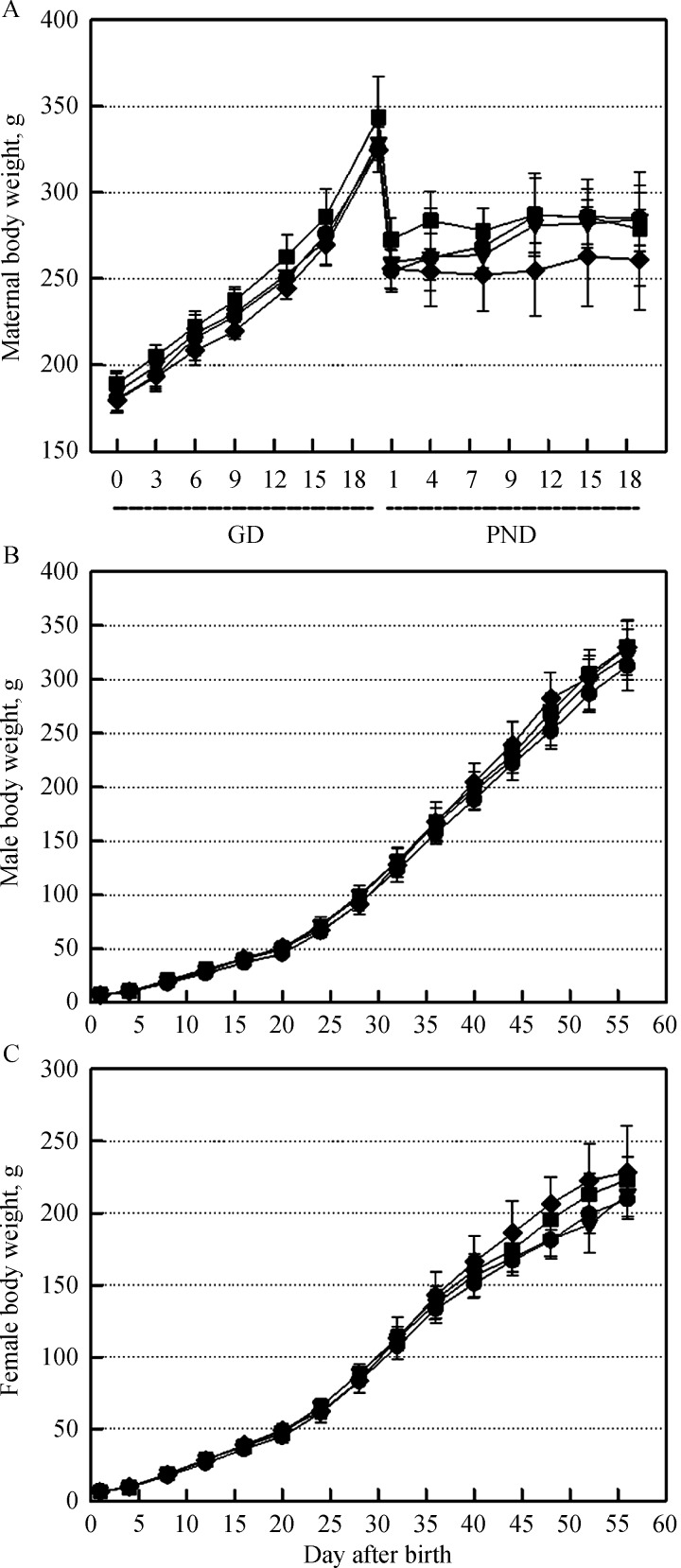
Changes in bodyweights of maternal rats and their offspring administered with 5% soybean milk (GD5%) from gestational day 6 (GD6) to parturition or 5% (GD-PND5%) and 100% (GD-PND100%) soybean milk from GD6 to postnatal day 56 (PND56), respectively.

In all animals in the control and soybean milk-treatment groups (GD5%: 5% soybean milk during gestational days, GD-PND5%: 5% soybean milk during gestational and post-natal days, GD-PND100%: 100% soybean milk during the gestational and post-natal days), no specific symptoms in dams and offspring were observed during the entire experimental period. The bodyweight gains of soybean milk-treated dams and offspring also displayed patterns similar to those of the control group (***Fig. 1***).

No significant differences in delivery indices, such as duration of pregnancy, stillbirth rate, and sex ratio in male to female neonates, were observed (***Table 1***). Furthermore, developmental parameters including the times of abdominal hair growth, incisor teeth eruption, eyelid opening, testicular descent, and vaginal opening in male and female offspring were not significantly different between control and soybean milk-treated rats.

**Tab.1:** Birth and physical development of offsprings born of maternal rats fed soybean milk

Gender	Development	Control	GD50%	GD-PND5%	GD-PND100%
Both	Duration of pregnancy (day)	21.71±0.46	21.67±0.48	22.00±0.05	21.71±0.46
	Stillbirth (%)	2.94	4.41	2.11	2.47
	Sex ratio (male : female)	53.0 : 47.0	51.9 : 48.5	45.2 : 55.8	50.6 : 49.4
	Appearance of abdominal hair (day)	7.05±0.23	6.85±0.41	6.85±0.41	7.04±0.54
	Incisor eruption (day)	10.80±0.52	10.94±0.52	10.89±0.57	10.96±0.72
	Eyelid opening (day)	15.43±0.57	15.23±0.59	14.91±0.49	15.24±0.64
Male	Testicular descent (day)	20.24±0.44	20.26±0.45	20.12±0.33	20.15±0.37
Female	Vaginal opening (day)	34.43±1.70	35.55±2.07	34.29±1.73	32.43±2.06

GD: gestational day, PND: postnatal day.

**Fig.2:**
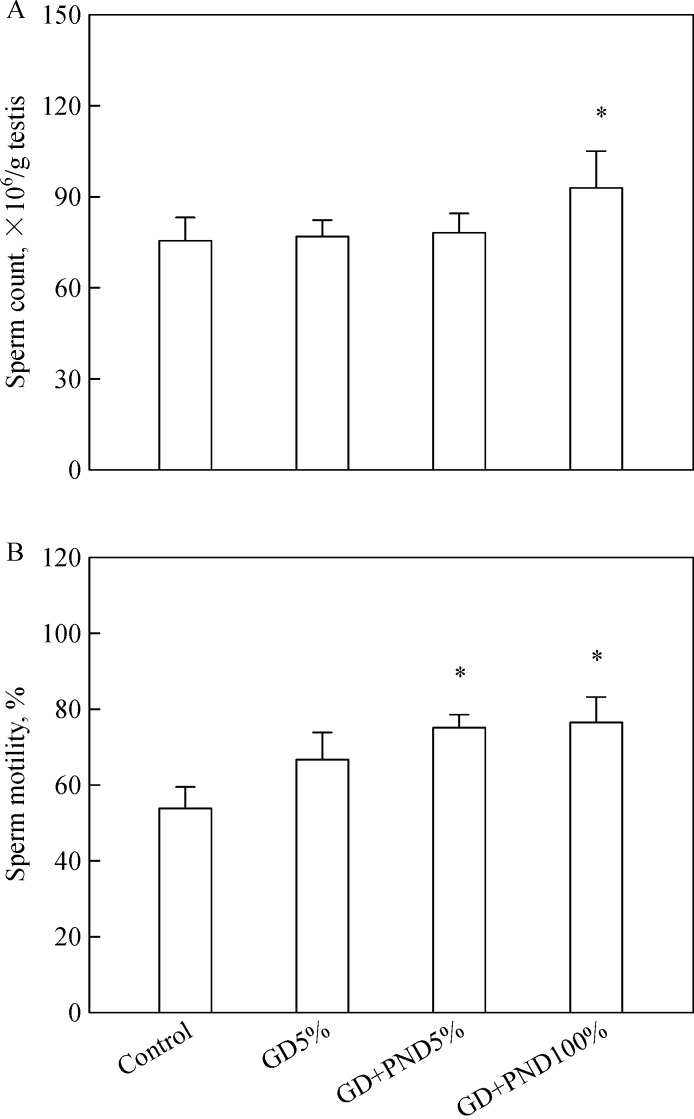
Microscopic findings of the reproductive organs of male offspring administered with 100% (GD-PND100%) soybean milk from gestational day 6 (GD6) to postnatal day 56 (PND56).

In the reproductive organ weight, the weights of testes and epididymides at PND57 tended to increase following soybean milk feeding: i.e., a long-term treatment (GD-PND) of 100% soybean milk, instead of drinking water, significantly increased both organs, although 5% soybean milk did not significantly increase their weights (***Table 2***). Soybean milk did not affect the weights of the prostates and seminal vesicles in males, nor the uterus and ovaries in females. By comparison, feeding soybean milk only during GD was ineffective in increasing both the male and female reproductive organ weights. In microscopic findings, no abnormal features were observed in the testes, epididymides, prostates, and seminal vesicles of male offpring exposed to soybean milk during GD-PND periods (***Fig. 2***).

**Tab.2:** Reproductive organ weights (g) of offsprings born of maternal rats fed soybean milk

Gender	Organ	Control	GD50%	GD-PND5%	GD-PND100%
Male	Testes	3.07±0.14	3.06±0.20	3.18±0.16	3.38±0.11*
	Epididymides	0.57±0.07	0.60±0.05	062±0.06	0.69±0.11*
	Prostates	0.28±0.05	0.28±0.04	0.32±0.06	0.32±0.05
	Seminal vesicles	0.92±0.23	0.94±0.10	0.99±0.19	1.11±0.13
Female	Uterus	0.34±0.05	0.33±0.03	0.39±0.11	0.40±0.17
	Ovaries	0.108±0.048	0.112±0.045	0.113±0.035	0.103±0.036

*Significantly different from control (*P*<0.05). GD: gestational day, PND: postnatal day.

**Fig.3:**
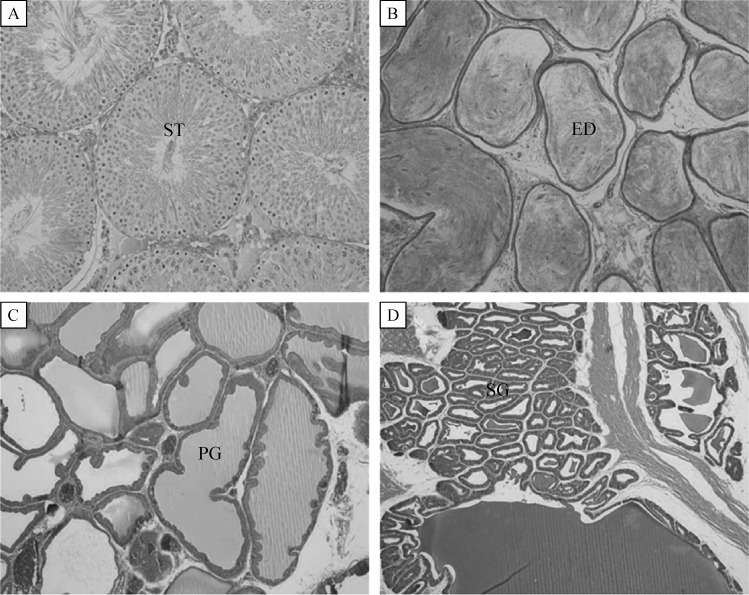
Changes in sperm counts (A) and motility (B) of male offspring administered with 5% soybean milk (GD5%) from gestational day 6 (GD6) to parturition or 5% (GD-PND5%) and 100% (GD-PND100%) soybean milk from GD6 to postnatal day 56 (PND56), respectively.

Notably, the sperm count significantly increased (by 23%) following feeding 100% soybean milk during both the GD and PND periods (GD-PND100%, ***Fig. 3A***). In addition, sperm motility also enhanced to 76.6% in the GD-PND100% group from 54.1% in the control animals (***Fig. 3B***). A low concentration (5%) of soybean milk also enhanced sperm motility, although no statistical significance after feeding only during the GD period was reached.

## Discussion

In the present study, ingestion of a soybean milk containing phytoestrogens during gestational and post-natal days (from GD6 to PND56) remarkably improved male reproductive function, in which weights of the testes and epididymides, as well as sperm count and motility, significantly increased. Indeed, based on the 10% and 21% increases in the testes and epididymides weights, respectively, the total sperm number in the GD-PND100% group increased by 26%, in addition to the increase in sperm motility. Therefore, it is of interest to note that a long-term feeding of soybean milk exerts beneficial effects on the post-generational male reproductive function.

In spite of the beneficial efficacies of soy isoflavones for menopausal symptoms^[3]^, there have been controversial results on the endocrine system due to the estrogenic activity of aglycones including genistein, daidzein, and glycitein. However, it is clear that there are different effects of isoflavones on the male reproductive system according to the exposure time during various developmental periods. High doses of isoflavones (up to 2,000 ppm in diet) and genistein (up to 1,250 ppm in diet) did not cause any adverse effects on adult and neonatal male rats^[19– 20]^. By contrast, genistein (higher than 1,250 ppm) decreased the birthweight and delayed development of rat offspring with aberrant spermatogenesis following exposure during the GD-PND periods^[21– 22]^ and increased the developmental toxicity of phthalate^[23]^, implying that gonadal development during the gestational period may be affected by isoflavones. Furthermore, it is widely assumed that the adverse effects of isoflavones on the spermatogenesis are prevented by blood-testis barrier which may be incomplete for permeability during the GD and neonatal periods. Indeed, it was reported that genistein and genistin were cytotoxic to cultured myogenic cells, wherein inhibition of protein synthesis and cell proliferation was observed as low as 1 mmol/L^[24]^. It was also demonstrated that soy isoflavones altered transcription levels of important proteins in Sertoli cell culture^[25]^, and that phytoestrogens kudzu and puerarin reduced motility and acrosome reaction of boar spermatozoa *via* ERβ *in vitro* without barrier^[26]^. In addition, genistein inhibited steroidogenesis *via* ERα and thereby impaired testosterone secretion from E12.5 fetal testis culture, but not from E18.5 testis^[27]^, suggesting that there is a susceptible period in embryo-fetal stages as well. Otherwise, glycosides including genistin, daidzin, and glycitin were major isoflavones in Vegemil^®^, in comparison with relatively-low contents of estrogenic aglycone isoflavones. Therefore, it is assumed that Vegemil^®^ did not exert adverse effects found from genistein exposure.

It is of interest to note that active aromatase and ER are found in immature germ cells and ejaculated spermatozoa, as well as in Leydig cells and Sertoli cells. Aromatase, a converting enzyme of testosterone to estrogen, is upregulated in motile spermatozoa, and estrogen produced locally is required for spermatogenesis, spermiogenesis, and sperm motility^[28– 31]^. In comparison with predominant expression of androgen receptors (AR) in somatic cells, ER was localized in testicular cells, indicative of the role of estrogen in the regulation of spermatogenesis (proliferation, apoptosis, survival, and maturation) and acquisition of motility^[31– 32]^. Interestingly, it was demonstrated that high concentrations (up to 1,000 ppm) of genistein did not inhibit aromatase, in contrast to a strong inhibition by diethylstilbestrol (DES), and that soy meal protected germ cells in aromatase-knockout mice displaying spermatogenic impairment^[33]^. Actually, another phytoestrogen *trans*-resveratrol increased gonadotrophins and sperm counts in rats^[34– 35]^. Recently, Eumkeb *et al*. reported that daidzein and genistein, extracted from *Butea superb* Roxb., which has been used as an aphrodisiac to improve erectile dysfunction in humans, increased testosterone level, as well as sperm number and motility in mice^[36]^. In line with the above reports, our results also demonstrate that soybean milk containing isoflavones enhance sperm count and motility in rats.

Collectively, the dose-response relationship in the beneficial and adverse effects of phytoestrogens on male reproductive function might be dependent on their accessibility to germ cells in the seminiferous tubules, although there may be species-specific differences^[37]^: i.e., exposure to high concentrations of isoflavones during the embryo-fetal stages may affect the spermatogenic process. In mice, soy-based products (340 ppm isoflavones) administered from conception to adulthood (GD-PND) impaired spermatogenesis, resulting in reduced epididymal sperm counts and litter size of offspring^[38]^. In addition, administration of a low dose (5 mg/kg ≒ 62.5 ppm) of genistein to gestational and lactational mouse dams slightly altered sperm counts and motility of offspring^[7]^. On the contrary, a high concentration (600 ppm) of soy/isoflavone-rich diet fed during the GD-PND periods enhanced testicular and prostate health in rats^[39]^. These findings are consistent with our observations that feeding rats with soybean milk (containing 8.1-162 ppm isoflavones) increased sperm counts and motility. Actually, the critical dose of genistein for embryotoxicity was suggested to be 100 mg/kg (1,250 ppm) in rats^[21– 22, 40]^.

Estrogens play a significant role in sperm development by affecting the transcriptional process of target genes through ERα or ERβ^[30– 32]^. In animals and humans deficient in ER or aromatase, the number and motility of sperms decreased, leading to infertility^[33, 41]^. In the present and previous studies, it was demonstrated that feeding soybean milk during the GD-PND periods improved the male reproductive function, and that soy meal protected germ cells in mice with spermatogenic dysfunction^[33]^. In human studies, soy-based infant formula did not lead to considerable differences in the developmental, endocrinological and reproductive outcomes in young adulthood^[42– 43]^.

The results indicate that soybean milk containing relatively-low concentrations of glycoside isoflavones is safe for dams, embryos, fetuses, and offspring, as well as improves the post-generational development of the male reproductive function. Although optimal concentrations for the beneficial effects of phytoestrogens remain to be clarified, it is also suggested that formulas containing phytoestrogens, such as isoflavones and *trans*-resveratrol, could be good strategies for the improvement of male reproductive function of infants and/or infertile men.
